# Neural-Network Quantum States for Spin-1 Systems: Spin-Basis and Parameterization Effects on Compactness of Representations

**DOI:** 10.3390/e23070879

**Published:** 2021-07-09

**Authors:** Michael Y. Pei, Stephen R. Clark

**Affiliations:** H.H. Wills Physics Laboratory, University of Bristol, Bristol BS8 1TL, UK

**Keywords:** neural-network quantum states, tensor network theory, restricted boltzmann machines

## Abstract

Neural network quantum states (NQS) have been widely applied to spin-1/2 systems, where they have proven to be highly effective. The application to systems with larger on-site dimension, such as spin-1 or bosonic systems, has been explored less and predominantly using spin-1/2 Restricted Boltzmann Machines (RBMs) with a one-hot/unary encoding. Here, we propose a more direct generalization of RBMs for spin-1 that retains the key properties of the standard spin-1/2 RBM, specifically trivial product states representations, labeling freedom for the visible variables and gauge equivalence to the tensor network formulation. To test this new approach, we present variational Monte Carlo (VMC) calculations for the spin-1 anti-ferromagnetic Heisenberg (AFH) model and benchmark it against the one-hot/unary encoded RBM demonstrating that it achieves the same accuracy with substantially fewer variational parameters. Furthermore, we investigate how the hidden unit complexity of NQS depend on the local single-spin basis used. Exploiting the tensor network version of our RBM we construct an analytic NQS representation of the Affleck-Kennedy-Lieb-Tasaki (AKLT) state in the xyz spin-1 basis using only M=2N hidden units, compared to $M∼O(N^{2})$ required in the $S^{z}$ basis. Additional VMC calculations provide strong evidence that the AKLT state in fact possesses an exact *compact* NQS representation in the xyz basis with only M=N hidden units. These insights help to further unravel how to most effectively adapt the NQS framework for more complex quantum systems.

## 1. Introduction

The strongly-interacting quantum many-body problem is crucial to our understanding of many intriguing physical phenomena, but it is also inherently difficult to treat numerically owing to the exponential growth of the Hilbert space with system size. A commonly used approximate strategy is the variational method where a trial state, characterized by a tractable number of variational parameters, is optimized in energy. The effectiveness of this approach is highly dependent on the ansatz having an expressive form that can be systematically improved, to minimize bias, while also allowing relevant observables to be evaluated efficiently. Tensor networks have provided several examples of such ansatzes, with matrix product states (MPS) [1,2] displaying impressive accuracy in one-dimensional systems, along with projected entangled-pair states (PEPS) [1,3], tree tensor networks (TTN) [4,5], and multi-scale entanglement renormalization ansatz (MERA) [6] making two-dimensional systems accessible. Recently, artificial neural networks (ANNs) have emerged as another class of highly flexible variational ansatzes with many variants, such as restricted Boltzmann machines (RBM) [7], Deep Boltzmann Machines (DBM) [8,9,10], convolutional neural networks (CNN) [11,12,13,14,15], and feed-forward neural networks (FFNN) [16,17,18,19]. An important advantage of ANNs is that they are highly flexible and can be applied to any number of spatial dimensions, making them a powerful method for tackling the subtle physics seen in two-dimensional systems.

Although one of the simplest ANN variants, RBMs have seen widespread applications, including for open quantum systems [20,21,22,23], frustrated spin problems [24,25], quantum circuit simulation [26,27,28], and more. There are several reasons for their continued use. First, their simple structure allows for efficient sampling crucial for applying variational Monte Carlo (VMC) [29,30]. Second, RBMs are also a good candidate for a weakly biased ansatz, given that they are capable of exactly representing arbitrary states when their hidden unit number *M* scales exponentially with system size *N*. Third, RBMs are capable of representing states with volume-law entanglement [31], which further distinguishes them from tensor networks [32], despite their conceptual similarities [33,34,35]. Finally, there are also numerous classes of states with efficient exact RBM representations, including graph states [8]; spin Jastrow states, such as Laughlin states [33,36,37]; and general stabilizer states, such as the toric code [38,39,40,41], as well as more exotic hypergraph states and XS-stabilizer states [40]. Recently, we found that all but the last class listed, in fact, have RBM representations requiring M<N hidden units [42], illustrating how even very modestly sized RBMs have significant representational power.

Despite their efficacy for spin-$\frac{1}{2}$ systems, the application of RBMs to systems with a local on-site dimension d>2, such as spin-1 or bosonic systems, has been limited with convolutional or feedforward neural networks generally being favored [16,17,43]. The typical approach in machine learning to handle models with multinomial or categorical variables is so-called “one-hot” or “unary” encoding [44]. Rather than representing a physical degree of freedom directly with one visible unit this approach encodes the possible local physical states into a set of binary visible units. While this approach leverages the power of binary or spin-$\frac{1}{2}$ RBMs, it multiplies the number of visible units by a factor *d*, significantly increasing the parameter count and complexity of the optimization. As a consequence, studies utilizing unary encoding so far, for example, on the Bose Hubbard model [45,46], have been limited to small system sizes. Thus, there is need to devise more efficient RBM constructions tailored for d>2 systems.

Progress has been made in this direction in recent work [47], where multivalued RBMs were applied directly to the one-dimensional spin-1 anti-ferromagnetic Heisenberg model (AFH) and substantially enhanced by incorporating a transformation to a coupled SU(2) symmetric basis. Complementary to this, here, we propose and study a direct generalization of the RBMs to spin-1 systems that retains key properties of spin-$\frac{1}{2}$ RBM with a minimal increase in variational parameters (as we will not be examining other network architectures, we will use RBM and NQS interchangeably in this paper). Specifically, the ability to describe arbitrary product states without hidden units, invariance of the parameterization to the values assigned to visible variables (labeling freedom), and equivalence to the tensor network formulation. This leads to the introduction of new quadratic bias and interaction weights in the RBM effective energy function. We demonstrate the effectiveness of the new formulation via VMC calculations for the spin-1 AFH model, where it is seen to deliver the same accuracy as unary encoding but with substantially fewer variational parameters. Additionally, we also investigate how the local single-spin basis affects the hidden unit complexity of a state by performing calculations in both the $S^{z}$ and xyz spin-1 bases. For the AFH model with periodic boundary conditions, we find that the $S^{z}$ is more accurate. A useful advantage of our new spin-1 RBM formulation is that it permits tensor network based analytic constructions. Focusing on the paradigmatic Affleck-Kennedy-Lieb-Tasaki (AKLT) model, we give explicit exact NQS representations in both the $S^{z}$ and xyz bases. Our $S^{z}$ basis NQS construction displays the expected [36] $M∼O(N^{2})$ scaling, while the simplification of the amplitude structure in the xyz basis gives an NQS construction with M=2N hidden units. By using VMC calculations, we find compelling evidence that the AKLT state, in fact, only requires M=N hidden units to be represented exactly in the xyz basis.

The structure of this paper is as follows. We briefly outline the VMC method applied to the many body problem in Section 2 and discuss desirable properties for variational ansatzes. Next, in Section 3, we discuss the RBM in its spin-$\frac{1}{2}$ form and analyze its key properties. In Section 4, we introduce a new generalization of NQS to spin-1 systems designed to mimic these properties and present VMC results for the AFH model. We then introduce analytic constructions for the AKLT model in Section 5, followed by VMC calculations in both the $S^{z}$ and xyz basis. Finally, in Section 6, we conclude and discuss some open problems.

## 2. The Many-Body Problem and Variational Monte Carlo

### 2.1. Quantum Many-Body Problem

In this work, we will focus on physical systems composed of *N* localized spinful particles. Each particle is described by a vector of spin operators ${\hat{S}}_{j}=\left({\hat{S}}_{j}^{x},{\hat{S}}_{j}^{y},{\hat{S}}_{j}^{z}\right)$ for j=1,2,⋯,N. Typically, the eigenstates $|\;S_{j}\rangle$ of ${\hat{S}}_{j}^{z}$ are used as the local spin basis, from which the $S^{z}$ basis for the full system is constructed as $|\;S\rangle =|\;S_{1}\rangle ⊗\cdots ⊗|\;S_{N}\rangle$, where $S=(S_{1},S_{2},\cdots ,S_{N})$. Any many-body quantum state for these *N* spins can then be expanded in this basis as

(1) $$|\;\Psi \rangle =\sum_{S}\Psi \left(S\right)|\;S\rangle ,$$

via its complex amplitudes Ψ(S). In the spin-$\frac{1}{2}$ case, we have ${\hat{S}}_{j}^{\alpha }=\frac{1}{2}{\hat{\sigma }}_{j}^{\alpha }$ (taking ℏ=1 throughout) for α={x,y,z} defined by the Pauli matrices, and $S_{j}\in \{+\frac{1}{2}\equiv \;↑,-\frac{1}{2}\equiv \;↓\}$. In the spin-1 case, we have

$$\begin{array}{ccc} \;{\hat{S}}_{j}^{x} & = & \frac{1}{\sqrt{2}}\left[\begin{array}{ccc} 0 & 1 & 0\\ 1 & 0 & 1\\ 0 & 1 & 0 \end{array}\right],\;{\hat{S}}_{j}^{y}=\frac{1}{\sqrt{2}}\left[\begin{array}{ccc} 0 & -i & 0\\ i & 0 & -i\\ 0 & i & 0 \end{array}\right],\;{\hat{S}}_{j}^{z}=\left[\begin{array}{ccc} 1 & 0 & 0\\ 0 & 0 & 0\\ 0 & 0 & -1 \end{array}\right], \end{array}$$

with $S_{j}\in \left\{+1\equiv \;⇑,0,-1\equiv \;⇓\right\}$.

A key challenge in many-body physics is to find the ground state $|\;\Psi_{0}\rangle$ of a system governed by an interacting Hamiltonian $\hat{H}$. In the context of spin systems, $\hat{H}$ will include terms that are products of the spin operators ${\hat{S}}_{j}$ over two or more spins. Given that the expectation value of any observable $\hat{A}$ for a general (unnormalized) state $|\;\Psi \rangle$ is

(2) $${\langle A\rangle }_{\Psi }=\frac{\langle \Psi \;|\hat{A}|\;\Psi \rangle }{\langle \;\Psi \;|\;\Psi \;\rangle },$$

the variational approach reformulates the eigenvalue problem $\hat{H}|\;\Psi_{0}\rangle =E_{0}|\;\Psi_{0}\rangle$ as the minimization of the energy $E={\langle \hat{H}\rangle }_{\Psi }$ over the exponentially many amplitudes Ψ(S). Performing this task exactly is only feasible for small systems N∼O(10) [48].

### 2.2. Variational Monte Carlo Method

A way to circumvent the “curse of dimensionality” is to instead to restrict the optimization over a specialized class of states $|\;\Psi_{p}\rangle$, dependent on parameters p whose number $n_{\mathrm{params}}$ scales polynomially with *N*. The variational principle $E_{0}\leq E_{p_{0}}=\min_{p}{\langle \hat{H}\rangle }_{\Psi_{p}}$ then provides a route to finding the best approximation $|\;\Psi_{p_{0}}\rangle$ within the ansatz for $|\;\Psi_{0}\rangle$. The flexibility and utility of variational ansatz are greatly enhanced if instead of computing expectation values ${\langle A\rangle }_{\Psi_{p}}$ exactly we evaluate them approximately using Monte Carlo sampling. This approach is called *variational Monte Carlo* (VMC) and is described in detail in Appendix A. A key feature of VMC is that only ratios of amplitudes for an ansatz $\Psi_{p}\left(S\right)/\Psi_{p}\left(S^{\prime }\right)$ between different spin states $|\;S\rangle$ and $|\;S^{\prime }\rangle$ are needed within the algorithm allowing us to ignore the normalization of quantum states in this work. To be efficient, thus, we require that these amplitude ratios for our ansatz can be evaluated with an effort scaling polynomially with *N*.

All numerical calculations presented in this paper employ the powerful stochastic reconfiguration method [30,49] for optimizing the parameters p. While ansatzes with a larger number of parameters may describe more varied amplitude structure, in principle allowing greater accuracy, the effort to optimize them can scale as $O(n_{\mathrm{params}}^{3})$. It is, therefore, crucial that the functional form of any candidate ansatz $|\;\Psi_{p}\rangle$ is judicious in its use of variational parameters to avoid excessive redundancies.

## 3. Neural-Network Quantum States in Spin-1/2

In this section, we review NQS in terms of RBMs, discussing their form from both an energy function and tensor network perspective. In doing so, we highlight key properties of NQS that are desirable for expressiveness and the ability to represent important families of states.

### 3.1. Restricted Boltzmann Machine Approach

Restricted Boltzmann machines consist of two sets of units, *Nvisible* units representing the physical system, and *Mhidden* units to be marginalized out. The units are characterized by a Boltzmann-like combined probability distribution $p\left(v,h\right)=\exp\left(-E_{\lambda }\right)$ with an effective “energy” function

(3) $$E_{\lambda }\left(v,h\right)=-\sum_{j=1}^{N}a_{j}v_{j}-\sum_{i=1}^{M}b_{i}h_{i}-\sum_{i=1}^{M}\sum_{j=1}^{N}w_{ij}h_{i}v_{j},$$

where λ={a,b,w} is the set of MN+M+N complex parameters consisting of *N* visible biases $a=(a_{1},\cdots ,a_{N})$, *M* hidden biases $b=(b_{1},\cdots ,b_{M})$, and M×N hidden-visible interaction weights $w=[w_{11},\cdots ,w_{1N},\cdots ,w_{MN}]$. While p(v,h) is Boltzmann once the hidden units are traced out, we are left with a more complex marginal distribution for the visible units, from which the NQS amplitudes are derived [7]:

(4) $$\Psi_{\lambda }\left(S\mapsto v\right)=\sum_{h}\exp\left[-E_{\lambda }\left(v,h\right)\right].$$

**Figure 1:**
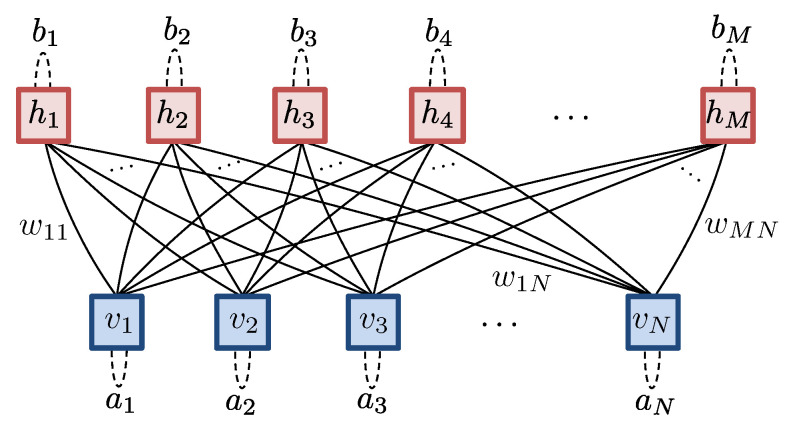
The bipartite graph of an RBM depicting interaction weights w as edges shown as solid arcs between hidden and visible units. For completeness, the biases a and b on each unit are depicted here as self-loop edges with dotted arcs to distinguish them from the interactions. The diagram here has been duplicated from ref. [42] and is presented here again for clarity.

The RBM architecture is shown diagrammatically in Figure 1.

Typically, visible $v_{j}$ and hidden $h_{i}$ unit variables are taken as two-valued. The pair of unique values {μ,ν} taken by units within the energy function Equation (3) can be freely chosen, and they need not coincide with the eigenvalues $S_{j}\in \left\{+\frac{1}{2},-\frac{1}{2}\right\}$ of the local operator ${\hat{S}}^{z}$ chosen to define the physical basis. In Equation (4), we emphasize this *labeling freedom* by explicitly introducing a mapping between physical and visible configuration S↦v. Commonly, with NQS, an implicit choice v=S is made, nullifying the need for this distinction, but it will prove to be useful here. Canonical choices $v_{j}$ are number-like {0,1} or Ising-like {+1,−1} values. We will adopt Ising-like visible variables, in which case it follows that an arbitrary visible unit taking values

(5) $$S_{j}=\left\{\begin{array}{ccc} ↑ & \mapsto & \mu \\ ↓ & \mapsto & \nu \end{array}\right\}={\tilde{v}}_{j}$$

is generated by a shift and rescaling of $v_{j}\in \left\{+1,-1\right\}$ as ${\tilde{v}}_{j}=\frac{1}{2}\left(\mu +\nu \right)+\frac{1}{2}\left(\mu -\nu \right)v_{j}$. Since this is a linear transformation, it is entirely accommodated by redefining the energy function $E_{\lambda }\left(v,h\right)$ parameters λ without changing its functional form. We shall see shortly that labeling freedom is a crucial ingredient for generalizing RBMs to spin-1 system using higher-dimensional visible units in Section 4. Tracing out Ising-like hidden variables results in the amplitude expansion [7]

(6) $$\begin{array}{ccc} \Psi_{\lambda }\left(S\mapsto v\right) & = & \prod_{j=1}^{N}e^{a_{j}v_{j}}\prod_{i=1}^{M}2\cosh\left(b_{i}+\sum_{j=1}^{N}w_{ij}v_{j}\right), \end{array}$$

that is commonly used and numerically convenient.

A key property of spin-$\frac{1}{2}$ RBMs is that they can represent any generic product state of the form

(7) $$\left(c_{+}^{\left(1\right)}|\;↑\rangle +c_{-}^{\left(1\right)}|\;↓\rangle \right)⊗\cdots ⊗\left(c_{+}^{\left(N\right)}|\;↑\rangle +c_{-}^{\left(N\right)}|\;↓\rangle \right),$$

without hidden units by simply setting their visible biases to

(8) $$a_{j}=\frac{\ln\left(c_{+}^{\left(j\right)}\right)-\ln\left(c_{-}^{\left(j\right)}\right)}{\mu -\nu }.$$

Hidden units are, thus, necessary to describe entangled states; however, unlike tensor networks, there is no direct quantitative relation between them. Indeed, Ref. [31] reports that, for random NQS, adding hidden units actually decreases the amount of entanglement in a state. Nevertheless, increasing the number *M* of hidden units increases the expressiveness of the NQS, allowing more complex correlations within $\Psi_{\lambda }\left(S\mapsto v\right)$ to be encoded. Formally, the NQS ansatz can represent any arbitrary state exactly in the limit of $M\to 2^{N}$[50]. More usefully, there are important classes of states that possess highly accurate approximate or even exact NQS representations with an efficient scaling M∼poly(N)  [33,36,38,39,40,41]. A particularly tractable NQS hidden unit complexity is typified by the following: efinition

**Definition** **1** (Compact NQS). *States with an exact NQS representation where M≤N will be denoted as compact.*

As we demonstrated in ref. [42], important classes of state, including Jastrow, graph, and stabilizer states, all have compact NQS representations.

### 3.2. Tensor Network Approach

**Figure 2:**
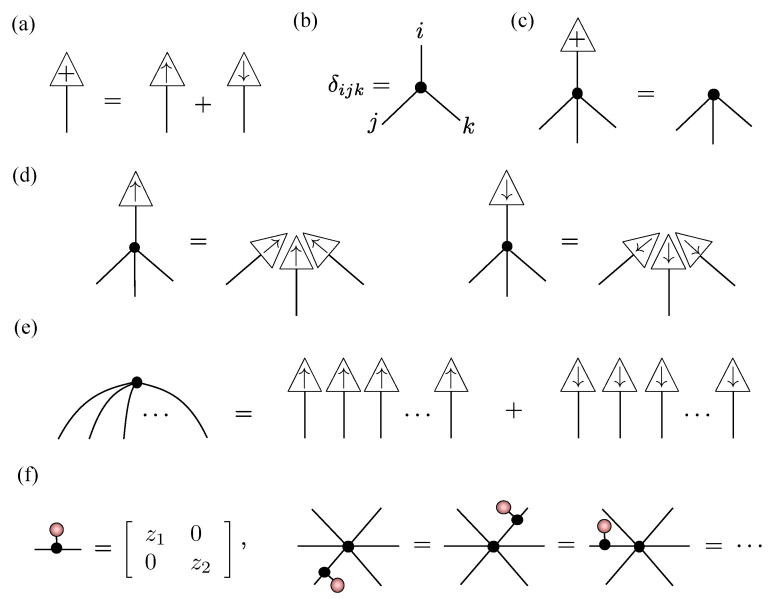
Useful spin-$\frac{1}{2}$ tensor network diagrams. (**a**) The $|\;+\rangle$ state. (**b**) An order-3 COPY tensor. (**c**) Termination of a COPY tensor leg with the $|\;+\rangle$ state. (**d**) Copying of basis state inputs by an order-4 COPY tensor. (**e**) Expansion of an order-*N* COPY tensor into a GHZ state. (**f**) The commutativity of diagonal order-2 tensors across a COPY tensor [42].

An alternative formulation of NQS views them as a tensor network. From this perspective, the amplitudes Ψ(S) are recast as elements of an order-*N* tensor $\Psi_{S_{1}S_{2}\cdots S_{N}}$, and this structureless tensor can be decomposed into a set of lower order tensors contracted together [1,2]. Here, we will make repeated use of tensor network diagrams that form an important analytical tool. Here, generic tensors of any order are represented as shapes, most often a circle ∘ color shaded to guide the eye, with protruding legs for each index they possess. Contraction of tensors is then represented by the joining of legs via graphical equations like this:(9)
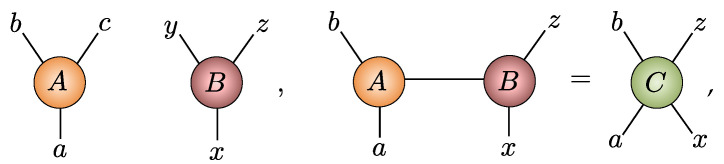

depicting $C_{abxz}=\sum_{\alpha }A_{ab\alpha }B_{x\alpha z}$. When there is a symbol inside a shape, it represents tensors with fixed elements or some specific structure. For example, the order-1 tensors with ↑ or ↓ symbols are the representation of the spin-$\frac{1}{2}$$S^{z}$ basis states ↑=(10) and ↓=(01), while the tensor shown in Figure 2a represents $|\;+\rangle =|\;↑\rangle +|\;↓\rangle$. A special tensor we will use frequently is the COPY tensor [33,51], denoted by a dot •, which is the multidimensional generalization of the 2×2 identity matrix. Its order-3 variant is shown in Figure 2b. It possesses a number of important properties. First, contracting any COPY tensor leg with $|\;+\rangle$ reduces the order of the COPY tensor by 1, as shown in Figure 2c. Second, contracting any COPY tensor leg with an $S^{z}$ basis state factorizes it by duplicating the basis vector across all legs, as shown in Figure 2d, motivating its name. This property makes COPY tensors a useful glue for constructing sampleable tensor networks. An order-*N* COPY tensor can itself be viewed as a representation of a GHZ state, shown in Figure 2e. Finally, Figure 2f shows a generic diagonal matrix attached to a leg of a COPY tensor can be commuted across to any of the COPY dot’s other legs.

With these concepts in place, the bipartite graph structure of RBM graph readily translates to a tensor network:(10)
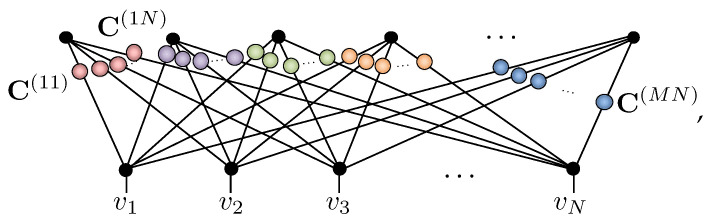

in which the vertices representing the visible and hidden units are replaced by COPY tensors, and order-2 tensors, or 2×2 coupling matrices $C^{\left(ij\right)}$, are introduced on each edge of the graph. Based on Figure 2e, thus, we can view each hidden unit in an NQS as contributing within an amplitude-wise product a locally deformed GHZ state. The NQS tensor network readily displays the key properties of the spin-$\frac{1}{2}$ RBM. They can trivially represent product states in Equation (7) by using one hidden unit with rank-1 coupling matrices P containing the coefficients $\left\{c_{+}^{\left(j\right)},c_{-}^{\left(j\right)}\right\}$ that subsequently factorizes out as
(11)
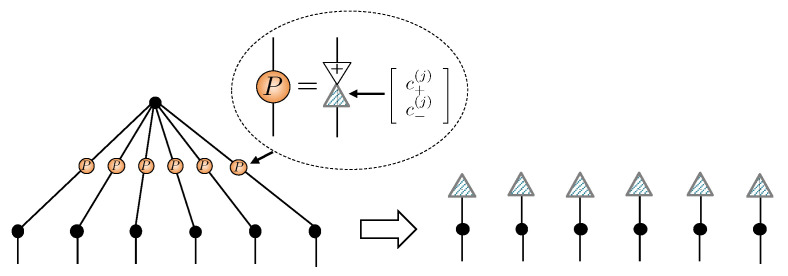
Moreover, since the elements of coupling matrices $C^{\left(ij\right)}$ have no functional dependence on any visible variables, they manifestly display labeling freedom. Crucially, while the NQS tensor network appears to have 4MN variational parameters, it exhibits significant gauge freedom due to the ability to reshuffle diagonal matrices across COPY tensors, as shown in Figure 2g. Consequently, most of the elements in the coupling matrices can be extracted and combined at the COPY tensors, reducing the number of independent parameters to N+M+NM, identical to the RBM formulation [42]. This equivalence allows either the NQS tensor network or RBM approach to be used interchangeably, and any direct generalization of RBMs to spin-1 should retain this feature.

## 4. Generalization to Spin-1 Systems

A tentative definition of a spin-1 RBM can be made by simply using the three-valued visible variables $v_{j}$ with a natural Ising-like physical to visible mapping,

(12) $$S_{j}=\left\{\begin{array}{ccc} ⇑ & \mapsto & +1\\ 0 & \mapsto & 0\\ ⇓ & \mapsto & -1 \end{array}\right\}=v_{j},$$

in the standard linear energy function Equation (3). By retaining two-valued Ising-like hidden units $h_{i}\in \left\{+1,-1\right\}$, the amplitudes $\Psi_{\lambda }\left(S\mapsto v\right)=\sum_{h}\exp\left[E_{\lambda }\left(v,h\right)\right]$ continue to be given by Equation (6), but now admitting three-valued visible variables.

This spin-1 generalization of the RBM lacks the key properties of the spin-$\frac{1}{2}$ variant. First, $\Psi_{\lambda }\left(S\mapsto v\right)$ cannot easily describe a generic product spin state

(13) $$\;\left(c_{+}^{\left(1\right)}|\;⇑\rangle +c_{0}^{\left(1\right)}|\;0\rangle +c_{-}^{\left(1\right)}|\;⇓\rangle \right)⊗\cdots ⊗\left(c_{+}^{\left(N\right)}|\;⇑\rangle +c_{0}^{\left(N\right)}|\;0\rangle +c_{-}^{\left(N\right)}|\;⇓\rangle \right),$$

with coefficients $c_{\alpha }^{\left(j\right)}$, since the visible bias term $\exp(a_{j}v_{j})$ cannot discriminate each of the local spin-1 states. This points to issues of expressiveness since we are using the same set of MN+M+N parameters {a,b,w} to describe a bigger state space. Second, the energy function Equation (3) does not possess labeling freedom for the visible units values. An arbitrary physical to visible mapping,

(14) $$S_{j}=\left\{\begin{array}{ccc} ⇑ & \mapsto & \mu \\ 0 & \mapsto & \nu \\ ⇓ & \mapsto & \omega \end{array}\right\}={\tilde{v}}_{j},$$

is generated from an Ising-like variable $v_{j}=\left\{+1,0,-1\right\}$ via the quadratic transformation ${\tilde{v}}_{j}=\nu +\frac{1}{2}\left(\mu -\omega \right)v_{j}+\frac{1}{2}\left(\mu +\omega -2\nu \right)v_{j}^{2}$. Consequently, transforming visible variables in this way cannot be accommodated in $E_{\lambda }\left(v,h\right)$ by changing only the parameters {a,b,w}. One way to avoid these issues is to use “one-hot” or “unary” encoding which has been successfully applied to both spin-1 [40] and bosonic systems [45,46].

### 4.1. Unary Encoding Approach

Unary encoding applies the spin-$\frac{1}{2}$ RBM formalism of Equation (4) to a larger state space of each spin-1 by mapping the physical system into one comprising a larger number of spin-$\frac{1}{2}$ particles. Specifically, the local $S^{z}$ basis of each spin-1 is mapped on to three spin-$\frac{1}{2}$’s as

(15) $$|\;⇑\rangle =|\;↑↑↓\rangle ,\;|\;0\rangle =|\;↑↓↑\rangle ,\;|\;⇓\rangle =|\;↓↑↑\rangle .$$

Physical states of the spin-1 system are now contained in the unary encoded subspace where only a single-excitation occurs within any three-site unit cell which facilitates the efficient projection of the representation. This naturally generalizes to a *d*-dimensional local state space.

By using this mapping, a spin-$\frac{1}{2}$ RBM can be applied inheriting its labeling freedom. Owing to the single-excitation projection, product states in Equation (13) are readily described by the visible biases $a_{ja},a_{jb}$ and $a_{jc}$ associated with the three spin-$\frac{1}{2}$’s {a,b,c} encoding a given spin-1. In general, for a *d*-dimensional local state space, unary encoding accounts for the enlarged state space by increasing the number of variational parameters to $n_{\mathrm{params}}=M+dN+dNM$ compared to the naive spin-1 RBM. However, there are some obvious deficiencies of this approach. First, unary encoding appears to unnecessarily enlarge the parameter count, as evidenced by the fact that it increases it even for d=2. This will significantly increase the computational cost. Second, splitting the interaction weights *w* across a unary cell makes the interpretation of any individual hidden unit’s contribution to the physical state rather opaque.

### 4.2. Defining a Spin-1 RBM and Tensor Network

These deficiencies show that a more general RBM energy function is needed that is sensitive to the three-valued nature of the visible units through the inclusion of terms involving the square of visible variables (a similar approach is likely to have been used in Ref. [47] already, although it was not explicitly stated). This motivates the following definition: efinition

**Definition** **2** (spin-1 NQS). *We introduce a direct spin-1 RBM as the ansatz with amplitudes $\Psi_{\Lambda }\left(S\mapsto v\right)=\sum_{h}\exp\left[E_{\Lambda }\left(v,h\right)\right]$ via the energy function*

(16) $$\begin{array}{c} \;E_{\Lambda }\left(v,h\right)=\sum_{j=1}^{N}a_{j}v_{j}+\sum_{j=1}^{N}A_{j}v_{j}^{2}+\sum_{i=1}^{M}b_{i}h_{i}+\sum_{i=1}^{M}\sum_{j=1}^{N}w_{ij}h_{i}v_{j}+\sum_{i=1}^{M}\sum_{j=1}^{N}W_{ij}h_{i}v_{j}^{2},\; \end{array}$$

*defined by the parameters Λ={a,b,w,W,A}.*

The new contributions to a spin-1 RBM are W, an M×N-dimensional matrix of quadratic interactions, and A, an *N*-dimensional vector of quadratic visible biases. There are now 2MN+2N+M complex parameters in total. Tracing out the two-valued Ising-like hidden units gives amplitudes

(17) $$\begin{array}{ccc} \;\Psi_{\Lambda }\left(S\mapsto v\right) & = & \prod_{i=1}^{N}e^{a_{i}v_{i}+A_{i}v_{i}^{2}}\prod_{j=1}^{M}2\cosh\left(b_{j}+\sum_{i=1}^{N}w_{ij}v_{i}+\sum_{i=1}^{N}W_{ij}v_{i}^{2}\right).\; \end{array}$$

The inclusion of a quadratic visible bias now allows any product state in Equation (7) to be described without hidden units by setting

$$a_{j}=\log\left(\sqrt{c_{+}^{\left(j\right)}c_{-}^{\left(j\right)}}/c_{0}^{\left(j\right)}\right)\;\mathrm{and}\;A_{j}=\log\left(\sqrt{c_{+}^{\left(j\right)}/c_{-}^{\left(j\right)}}\right),$$

while the quadratic interaction term ensures labeling freedom for the visible variable.

**Figure 3:**
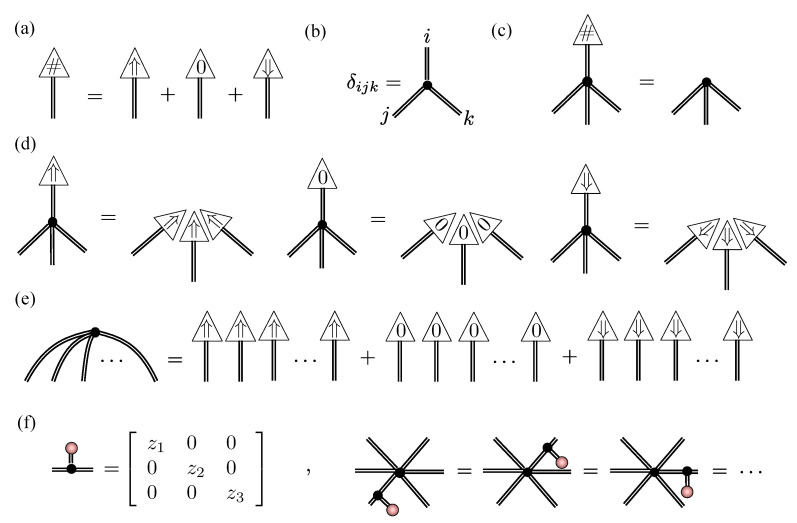
Useful spin-1 tensor network diagrams, directly generalizing the spin-$\frac{1}{2}$ properties outlined in Section 3.2. (**a**) The $|\;\#\rangle$ state. (**b**) An order-3 COPY tensor. (**c**) Termination of a COPY tensor leg with the $|\;\#\rangle$ state. (**d**) Copying of basis state inputs by an order-4 COPY tensor. (**e**) Expansion of an order-*N* COPY tensor into a spin-1 GHZ state. (**f**) The commutativity of diagonal order-2 tensors across a COPY tensor.

A strong justification for Equation (16) being the appropriate spin-1 generalization of RBMs is its relation to an NQS tensor network for spin-1. To handle spin-1 systems, we introduce a COPY tensor with three-dimensional legs copying the $S^{z}$ basis states $\left\{|\;⇑\rangle ,|\;0\rangle ,|\;⇓\rangle \right\}$. Its properties are summarized in Figure 3a–f and are straightforward generalizations of the spin-$\frac{1}{2}$ case given in Figure 2a–f and discussed in Section 3.2. The major difference is that we now distinguish three-dimensional legs with ‘=’ lines instead of ‘−’. As before, the NQS tensor network follows from the conversion of the RBM graph in Figure 1, except that visible vertices are now replaced by three-dimensional COPY tensors giving:(18)
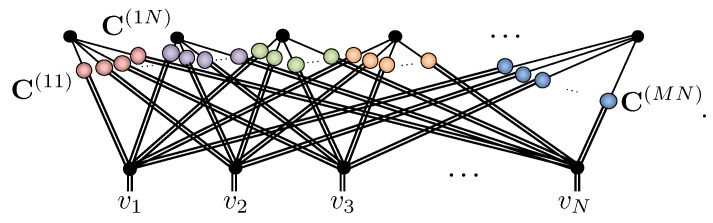
Owing to the mixed dimensionality of the COPY tensors in this network, it now requires a rectangular 2×3 coupling matrix $C^{\left(ij\right)}$ between the *i*-th hidden and *j*-th visible unit. For the *i*-th hidden unit, its set of coupling matrices $\Upsilon^{\left(i\right)}=\left\{C^{\left(i1\right)},C^{\left(i2\right)},\cdots ,C^{\left(iN\right)}\right\}$ can be explicitly tabulated as

$$\begin{array}{ccccc} \; & \overset{v_{1}}{\overbrace{+1\;\;\;\;0\;-1}} & \overset{v_{2}}{\overbrace{+1\;\;\;\;0\;-1}} & \cdots & \overset{v_{N}}{\overbrace{+1\;\;\;\;0\;-1}}\\ h_{i}\left\{\begin{array}{c} +1\\ -1 \end{array}\right. & \left[\begin{array}{ccc} C_{++}^{\left(i1\right)} & C_{+0}^{\left(i1\right)} & C_{+-}^{\left(i1\right)}\\ C_{-+}^{\left(i1\right)} & C_{-0}^{\left(i1\right)} & C_{--}^{\left(i1\right)} \end{array}\right] & \left[\begin{array}{ccc} C_{++}^{\left(i2\right)} & C_{+0}^{\left(i2\right)} & C_{+-}^{\left(i2\right)}\\ C_{-+}^{\left(i2\right)} & C_{-0}^{\left(i2\right)} & C_{--}^{\left(i2\right)} \end{array}\right] & \cdots & \left[\begin{array}{ccc} C_{++}^{\left(iN\right)} & C_{+0}^{\left(iN\right)} & C_{+-}^{\left(iN\right)}\\ C_{-+}^{\left(iN\right)} & C_{-0}^{\left(iN\right)} & C_{--}^{\left(iN\right)} \end{array}\right] \end{array}.$$

The amplitudes $\Upsilon^{\left(i\right)}\left(v\right)$ of this hidden unit’s correlator then follow by summing the product of coupling matrix elements selected by v along each row, giving

(19) $$\;\Upsilon^{\left(i\right)}\left(v\right)=\sum_{h_{i}=\pm 1}^{1}\prod_{j=1}^{N}C_{h_{j}v_{i}}^{\left(ij\right)}=C_{+v_{1}}^{\left(i1\right)}C_{+v_{2}}^{\left(i2\right)}\cdots C_{+v_{N}}^{\left(iN\right)}+C_{-v_{1}}^{\left(i1\right)}C_{-v_{2}}^{\left(i2\right)}\cdots C_{-v_{N}}^{\left(iN\right)}.$$

The amplitudes of NQS tensor network are then the product of each of these hidden unit correlators

(20) $$\begin{array}{ccc} \Psi_{\mathrm{NQS}}\left(S\to v\right) & = & \prod_{i=1}^{M}\Upsilon^{\left(i\right)}\left(v\right), \end{array}$$

which, like an RBM, can be exactly and efficiently sampled.

The spin-1 NQS tensor network appears to have 5MN complex variational parameters; however, again, gauge freedom allows the shuffling of diagonal matrices (of an appropriate dimension) through a COPY tensors reducing this. Specifically, its equivalence to the generalized spin-1 RBM proposed in Equation (16) is made using the coupling matrix decomposition

$$\begin{array}{ccc} \left[\begin{array}{ccc} C_{++}^{\left(ij\right)} & C_{+0}^{\left(ij\right)} & C_{+-}^{\left(ij\right)}\\ C_{-+}^{\left(ij\right)} & C_{-0}^{\left(ij\right)} & C_{--}^{\left(ij\right)} \end{array}\right] & = & e^{c}\left[\begin{array}{cc} e^{{\tilde{b}}_{ij}} & 0\\ 0 & e^{-{\tilde{b}}_{ij}} \end{array}\right]\left[\begin{array}{ccc} e^{w_{ij}+W_{ij}} & 1 & e^{-w_{ij}+W_{ij}}\\ e^{-w_{ij}-W_{ij}} & 1 & e^{w_{ij}-W_{ij}} \end{array}\right]\\ & & \;\;\;\;\;\times \left[\begin{array}{ccc} e^{{\tilde{a}}_{ij}+{\tilde{A}}_{ij}} & 0 & 0\\ 0 & 1 & 0\\ 0 & 0 & e^{-{\tilde{a}}_{ij}+{\tilde{A}}_{ij}} \end{array}\right]. \end{array}$$

The solution for the weights $w_{ij},W_{ij}$ and partial biases ${\tilde{a}}_{ij},{\tilde{A}}_{ij},{\tilde{b}}_{ij}$ is outlined in Appendix B, from which the full RBM biases are formed as $a_{j}=\sum_{i=1}^{M}{\tilde{a}}_{ij}$, $A_{j}=\sum_{i=1}^{M}{\tilde{A}}_{ij}$ and $b_{i}=\sum_{j=1}^{N}{\tilde{b}}_{ij}$. The spin-1 NQS tensor network, thus, reduces to $n_{\mathrm{params}}=2MN+2N+M$ complex parameters.

This correspondence between the spin-1 RBM and the spin-1 NQS tensor network highlights an advantage over unary encoding. Specifically, coupling matrices provide an intuitive tool for engineering the correlations and structures that a given hidden unit imprints on the amplitudes of an NQS. A trivial case is when all the elements of the *j*-th coupling matrix are 1’s, denoted generically as I, equivalent to the hidden unit being disconnected from that visible unit. A more complex example with conditional correlations is a hidden unit with coupling matrices

$$\{I,\cdots ,I,\overset{k}{\overbrace{C_{c⇑}}},C_{-0⇓},\cdots ,C_{-0⇓}\},$$

built from

$$C_{c⇑}=\left[\begin{array}{ccc} 0 & 1 & 1\\ 1 & 0 & 0 \end{array}\right]\;\mathrm{and}\;C_{-0⇓}=\left[\begin{array}{ccc} 1 & 1 & 1\\ 1 & -1 & -1 \end{array}\right].$$

This hidden unit generates a correlator $\Upsilon \left(v\right)=\delta_{v_{k},⇓}+\delta_{v_{k},⇑}{\left(-1\right)}^{C(v)}$, where a factor ${\left(-1\right)}^{C(v)}$ is introduced conditional on the *k*th spin being in the state $|\;⇑\rangle$, with C(v) being the number of $|\;0\rangle$ and $|\;⇓\rangle$ states in the configuration v between spins k+1 and *N*. Similar types of hidden units will be used extensively in Section 5.1 to construct an exact representation of a state.

### 4.3. Projection of Unary Encoding into a Spin-1 RBM

**Figure 4:**
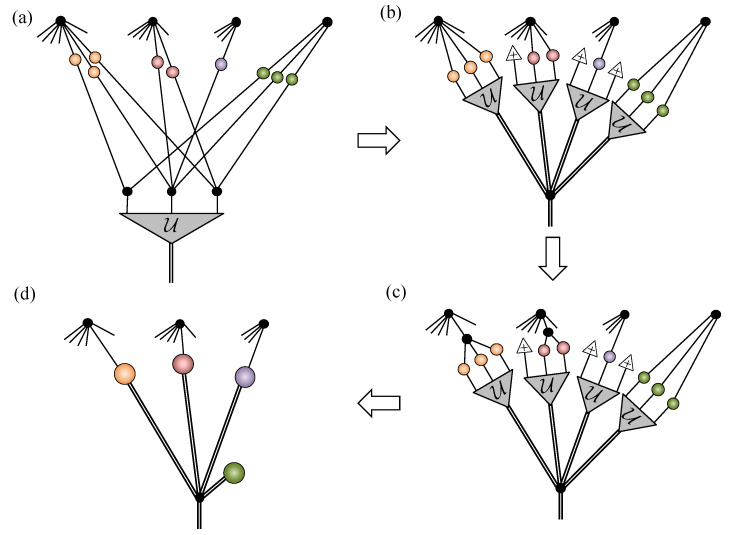
(**a**) Representative examples of unary projection contractions with hidden units possessing different patterns of connectivity. (**b**) The first step in the contraction involves pulling the projection through the two-dimensional COPY tensors, leaving behind a single three-dimensional COPY tensor. (**c**) Connections to the unary spins are isolated by splitting the hidden unit COPY tensors. (**d**) The resulting blocks of the network are then contracted to obtain the 2×3 spin-1 NQS coupling matrices.

The tensor network formalism provides further evidence that unary encoding from Section 4.1 is an over-parameterization of RBMs for spin-1 systems with $\delta n_{\mathrm{params}}=N\left(1+M\right)$ redundant parameters. Unary projection is implemented by an order-4 tensor U obeying:(21)
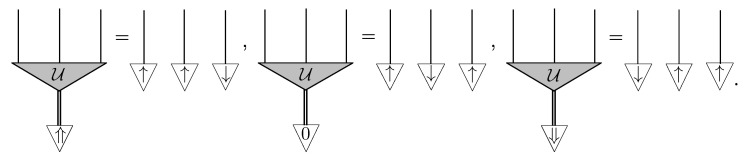
 By directly applying this projector to the spin-$\frac{1}{2}$ NQS tensor network for a unary encoded state and performing a graphical rewrite, we obtain the spin-1 NQS introduced in Equation (18). Figure 4 summarizes the crucial manipulations required.

In Figure 4a, some representative examples of contractions between the projection tensor U and hidden units are shown. There are three steps to rewriting the network. The first step, shown in Figure 4b, essentially pulls U through the three unary two-dimensional COPY tensors, leaving behind a single three-dimensional COPY tensor representing the physical spin-1. Two important cases are shown in the example in Figure 4b. A hidden unit may have connections to each of the unary spin-$\frac{1}{2}$’s, where upon they get bundled up by the U tensor. A hidden unit may connect to only a subset of the unary spin-$\frac{1}{2}$’s, which is handled by plugging the unused legs of U tensor with $|\;+\rangle$. If a hidden unit couples to more than one of the unary spins, then the second step, shown in Figure 4c, involves splitting the hidden unit’s two-dimensional COPY tensor to separate those connections. The final step is then to contract the split COPY tensor, coupling matrices and the projection U to form a rectangular 2×3 coupling matrix, as depicted in Figure 4d. If a hidden unit has connections exclusively within the unary spin-$\frac{1}{2}$’s, then it becomes an entirely local visible bias contribution in the spin-1 NQS.

### 4.4. Change of Local Spin Basis

For tensor network representations, such as MPS or PEPS, the complexity (internal bond dimension) of a given state’s description is rooted in its entanglement structure. As such changing the local basis of the spins used in a calculation has no effect on this complexity. Moreover, transforming a representation from one basis to another is accomplished by simply admixing the local tensors. Within VMC, a change of local spin basis leaves the locality and the sparsity of the Hamiltonian essentially unchanged. However, it is pivotal to the method that the amplitudes $\Psi_{p}\left(S\right)$ of whatever ansatz is used can be efficiently evaluated in this new basis. This is not generally true of NQS since their sampleability is intimately tied to the basis that factorizes the COPY tensors they are built from.

To understand how NQS behave, consider a representation of some state $|\;\Psi \rangle$, for instance, of four spin-1’s sampleable in the $S^{z}$ basis
(22)
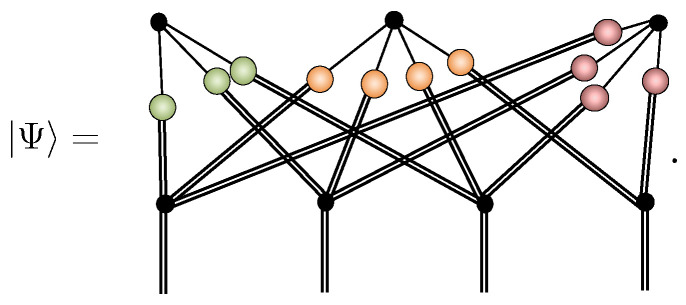
 Now, suppose we transform the local $S^{z}$ basis $|\;S\rangle$ to a new basis χ via a unitary $|\;\chi_{S}\rangle ={\hat{B}}^{†}|\;S\rangle$. Formally, from a tensor network perspective, we find the χ basis NQS representation by sandwiching $𝟙=\hat{B}{\hat{B}}^{†}$ on each physical leg and computing the $S^{z}$ basis NQS representation of ${\left({\hat{B}}^{†}\right)}^{⊗N}|\;\Psi \rangle$. However, currently, there is no known procedure for updating exactly an NQS after the application of an arbitrary single-spin unitary, even allowing for an increased number of hidden units. While we are guaranteed that an NQS representation of ${\hat{B}}^{⊗N}|\;\Psi \rangle$ exists, as illustrated here schematically
(23)
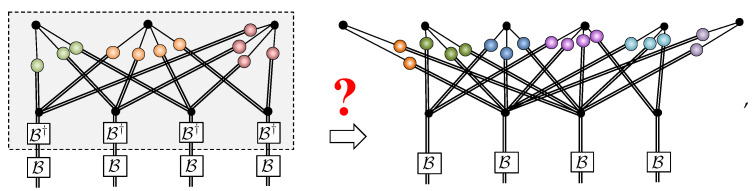

there is no guarantee it will be efficient, even if the representation of $|\;\Psi \rangle$ was originally. An example of such a catastrophic loss of NQS efficiency has been presented by Gao and Duan [8]. They show that, so long as the polynomial hierarchy in computational complexity theory does not collapse, a two-dimensional cluster state, which has an efficient NQS representation, has no efficient NQS representation after a specific layer of translation-invariant single-spin unitaries are applied. Thus, NQS complexity depends non-trivially on the local spin basis used.

Motivated by this, we will consider NQS calculations in two different local spin-1 bases to examine how the complexity varies, specifically, the standard $S^{z}$ basis and the xyz basis defined as

$$\begin{array}{c} |\;x\rangle =\frac{1}{\sqrt{2}}\left(|\;⇑\rangle -|\;⇓\rangle \right),\;|\;y\rangle =\frac{i}{\sqrt{2}}\left(|\;⇑\rangle +|\;⇓\rangle \right),\;|\;z\rangle =-|\;0\rangle . \end{array}$$

In the xyz basis, the individual spin-1 operators all acquire the same off-diagonal form

$$\begin{array}{ccc} \;{\hat{S}}_{j}^{x} & = & \left[\begin{array}{ccc} 0 & 0 & 0\\ 0 & 0 & i\\ 0 & -i & 0 \end{array}\right],\;{\hat{S}}_{j}^{y}=\left[\begin{array}{ccc} 0 & 0 & i\\ 0 & 0 & 0\\ -i & 0 & 0 \end{array}\right],\;{\hat{S}}_{j}^{z}=\left[\begin{array}{ccc} 0 & i & 0\\ -i & 0 & 0\\ 0 & 0 & 0 \end{array}\right], \end{array}$$

as a consequence of them all contributing one eigenstate to the basis. On a practical level, using the xyz basis for the spin-1 RBM in Equation (17) simply requires replacing S with α and a physical-visible mapping from α↦v, such as

(24) $$\alpha_{j}=\left\{\begin{array}{ccc} x & \mapsto & +1\\ y & \mapsto & 0\\ z & \mapsto & -1 \end{array}\right\}=v_{j}.$$

Equivalently, the visible unit COPY tensors for this NQS tensor network can be considered to be rotated into this xyz basis.

### 4.5. Numerical Example—Spin-1 Anti-Ferromagnetic Heisenberg Model

To confirm the effectiveness of our spin-1 NQS, we performed VMC calculations to reach the ground state of the well-known anti-ferromagnetic Heisenberg (AFH) model in one dimension. The Hamiltonian is given by

(25) $$\hat{H}=J\sum_{i}{\hat{S}}_{i}\cdot {\hat{S}}_{i+1},$$

where J>0 is the magnetic interaction strength and with periodic boundary conditions N+1≡1. We focus on small systems allowing direct comparison to the ground state calculated from exact diagonalization via the overlap $O=|\langle \;\Psi_{\mathrm{ED}}\;|\;\Psi_{\Lambda }\;\rangle {|}^{2}$. Additionally, we performed NQS optimizations in both the $S^{z}$ and xyz bases to compare any differences in performance. This basis change alters the fixed quantum numbers of the system. In particular, in the $S^{z}$ basis, the AFH model preserves the total $S^{z}$ projection of the system, and its ground state lies in the $\sum_{j=1}^{N}S_{j}^{z}=0$ sector of the full Hilbert space, while, in the xyz basis, the AFH model preserves the “parity” of the total x,y,z spin populations in the system, and, for even *N*, the ground state lies in the subspace of configurations, where there is an even number of each basis state. As is common for VMC calculations, we only select configurations from the relevant subspace during sampling. We perform the calculations with hidden unit numbers M=[1,2,⋯,2N], initializing M=1 with random small complex parameters. For each successive calculation, we need the NQS with the parameters for M−1 and initialize the *M*th hidden unit with random small parameters, gradually increasing the size of the network in a sequential manner. To check the robustness against initialization bias of the qualitative features we have discussed, we rerun optimization sequences 5 to 10 times and present the best results here.

**Figure 5:**
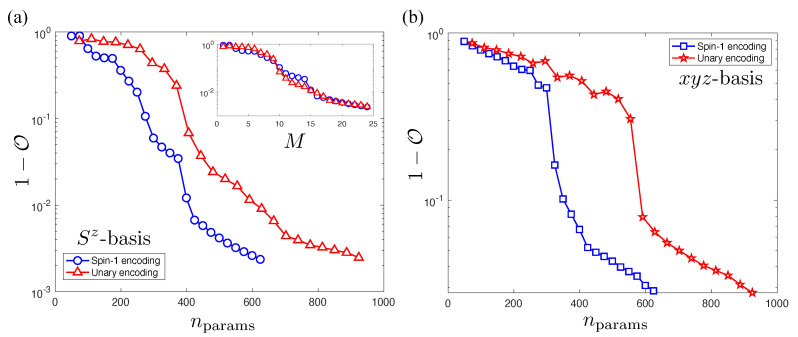
Plots of the infidelity of unary (red) and spin-1 NQS (blue) with the spin-1 AFH ground state with periodic boundary conditions. The calculations presented in both plots have the same number of sites N=12, up to M=2N=24 hidden units. (**a**) The infidelity 1−O of the two NQS formulations for the $S^{z}$ basis versus the RBM parameter count $n_{\mathrm{params}}$. The inset shows the collapse of the same data when it is plotted in terms of the hidden unit number *M*. (**b**) The same calculations performed in the xyz basis.

In Figure 5, we show how the accuracy of spin-1 NQS and unary encoding representations improve with an increasing number of hidden units *M* for both the $S^{z}$ and xyz bases plotted in terms of the variational parameter count. The spin-1 NQS achieves a superior accuracy to unary encoding for a similar $n_{\mathrm{params}}$. The inset of Figure 5a shows the collapse of the same $S^{z}$ data plotted against *M*, indicating that the spin-1 NQS and unary encoding have in fact located the same solution for a given *M*. However, by using $\delta n_{\mathrm{params}}=N\left(1+M\right)$ less parameters, the spin-1 NQS is considerably more efficient to optimize, especially noting that $\delta n_{\mathrm{params}}$ scales with both system size and hidden unit number (for example, consider that the N=12,M=12 spin-1 NQS has a comparable parameter count to an N=12,M=8 unary NQS). In Figure 5b, we observe a noticeable drop in accuracy for both NQS variants in the xyz basis compared to the $S^{z}$ basis. This suggests that the AFH ground state amplitude structure with periodic boundaries is inherently more complicated in the xyz basis regardless of encoding. Moreover, this confirms that hidden unit number *M* of an NQS is basis-dependent quantity and cannot be used as a proxy of the entanglement.

## 5. Revisiting the AKLT Model

We now move on to benchmark our spin-1 NQS against the analytically solvable AKLT model [52], which is a spin-1 chain governed by a bilinear-biquadratic SU(2)-isotropic Heisenberg Hamiltonian of the form

$${\hat{H}}_{\mathrm{AKLT}}=\sum_{j=1}^{N}\left[{\hat{S}}_{j}\cdot {\hat{S}}_{j+1}+\beta {\left({\hat{S}}_{j}\cdot {\hat{S}}_{j+1}\right)}^{2}\right]+\frac{2}{3},$$

with periodic boundary conditions N+1≡1. It has special significance since it was the first solvable spin-1 chain model that exhibits the ‘Haldane gap’ [53]. The AKLT state $|\;\Psi_{\mathrm{AKLT}}\rangle$ is the ground state of ${\hat{H}}_{\mathrm{AKLT}}$ at the AKLT point $\beta =\frac{1}{3}$, and has an energy of exactly zero.

**Figure 6:**
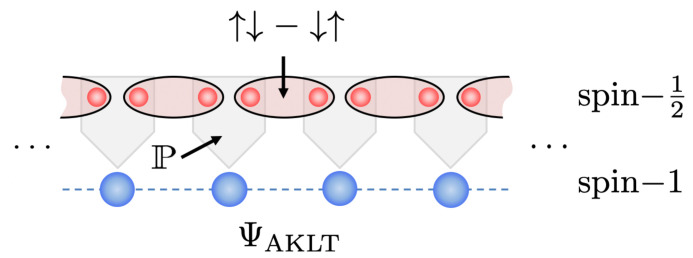
Valence bond solid construction of the spin-1 AKLT state $\Psi_{\mathrm{AKLT}}$ from the projection P of pairs of spin-$\frac{1}{2}$ particles shared between neighboring sites on the chain.

As is well-known, $|\;\Psi_{\mathrm{AKLT}}\rangle$ has a special structure of correlations which are related to a valence bond solid. Specifically, each spin-1 is envisaged as being a pair of spin-$\frac{1}{2}$ particles that are correspondingly entangled in a singlet state with a partner spin-$\frac{1}{2}$ in the nearest neighboring spin-1 on the chain. The AKLT state is then the projection P of the local pairs of spin-$\frac{1}{2}$ particles into the triplet subspace, as depicted in Figure 6. This also leads to $|\;\Psi_{\mathrm{AKLT}}\rangle$ possessing a very compact MPS representation with matrices

$$\begin{array}{ccc} A^{+} & = & \frac{1}{\sqrt{2}}{\hat{\sigma }}^{+},\;A^{0}=-\frac{1}{2}{\hat{\sigma }}^{z},\;A^{-}=-\frac{1}{\sqrt{2}}{\hat{\sigma }}^{-}, \end{array}$$

where ${\hat{\sigma }}^{\pm }=\frac{1}{2}\left({\hat{\sigma }}^{x}\pm i{\hat{\sigma }}^{y}\right)$, such that the (unnormalized) amplitudes of the ground state in the ${\hat{S}}^{z}$ basis follow as

(26) $$\Psi_{\mathrm{AKLT}}\left(S\right)=\mathrm{tr}\left(A^{S_{1}}A^{S_{2}}\cdots A^{S_{N}}\right).$$

Since the AKLT point of ${\hat{H}}_{\mathrm{AKLT}}$ lies in the gapped Haldane phase, $|\;\Psi_{\mathrm{AKLT}}\rangle$ has finite-ranged magnetic correlations,

(27) $$O_{\ell }^{zz}=\langle \psi_{\mathrm{AKLT}}\;|{\hat{S}}_{0}^{z}{\hat{S}}_{\ell }^{z}|\;\psi_{\mathrm{AKLT}}\rangle ∼e^{-\ell /\xi },\;\mathrm{with}\;\xi =\frac{1}{\ln(3)},$$

yet it also has an unbroken spin rotation symmetry which is a hallmark of a symmetry protected topological order. Specifically, the string-order parameter

(28) $$O^{\mathrm{string}}=\lim_{N\to \infty }\lim_{\ell \to \infty }\langle \psi_{\mathrm{AKLT}}\;|{\hat{S}}_{0}^{z}\prod_{j=1}^{\ell -1}e^{i\pi {\hat{S}}_{j}^{z}}{\hat{S}}_{\ell }^{z}|\;\psi_{\mathrm{AKLT}}\rangle ∼-\frac{4}{9},$$

reveals the presence of infinite-ranged anti-ferromagnetic correlations. This is evident from the structure of the MPS amplitudes. Any matrix product $A^{\pm }A^{0}\cdots A^{0}A^{\pm }=0$, so any configuration containing a ferromagnetic segment, like “+ 0 0 0 +”, with any number of 0’s is not allowed. In contrast, allowed configurations contain only anti-ferromagnetic segments, such as “– 0 + 0 0 0 – + 0”, arising from sequences, like $A^{\pm }A^{0}\cdots A^{0}A^{\mp }$, where every ± is partnered with a ∓ separated by with an arbitrary string of 0’s.

Despite its simple MPS representation it is surprisingly non-trivial to capture the non-commutative matrix products making up the AKLT amplitudes with an NQS. Direct conversion to an NQS from the MPS representation gives two reasons why it must contain long-ranged hidden units. First, it has been shown [38] that any short-ranged translationally invariant NQS cast into a MPS form by mapping hidden units into virtual bonds has A matrices that are at most rank-1. Since the matrix $A^{0}$ in the AKLT state is rank-2, it fails this condition. Second, if we divide the chain into a sequence of three contiguous parts a,b,c, once we make *b* larger than the longest range of any hidden unit, so no hidden unit connects to visible units in both *a* and *c*, then the NQS amplitudes factorize as

(29) $$\psi_{\mathrm{sr}-\mathrm{NQS}}\left(S_{a},S_{b},S_{c}\right)=\psi_{ab}\left(S_{a},S_{b}\right)\psi_{bc}\left(S_{b},S_{c}\right),$$

implying that $S_{a}$ and $S_{c}$ are uncorrelated [34]. The AKLT amplitudes $\Psi_{\mathrm{AKLT}}\left(S\right)$ do not satisfy this property since region *b* can be any length of 0’s, and there will always be non-zero amplitudes “+ 0 0 0 ...0 –” and “–  0 0 0 ...0 +” encoding string order correlations that are not factorizable.

It has been previously found that the AKLT state in the $S^{z}$ basis requires an NQS with $M∼O(N^{2})$ long-ranged hidden units [36], and this was borne out in numerical calculations for small systems. In Appendix C, we explicitly construct an NQS for the $S^{z}$ basis AKLT amplitudes using $M=2N^{2}+N\left\lfloor \frac{1}{2}\left(N-1\right)\right\rfloor +1$ hidden units, many of which are extensive over the system. The $O(N^{2})$ scaling can be readily understood as a consequence of having hidden units that each eliminate disallowed configurations, such as “± 0 0 0 ±”, and impose the sign for allowed configurations, such as “± 0 0 0 ∓”, for all *N* separations and *N* translations over the system. This is rather less efficient than the compact spin-$\frac{1}{2}$ NQS found for Jastrow, graph and stabilizer states in ref. [42]. The AKLT state can be expressed with O(N) hidden units but at the expense of needing a 2-layer DBM network [38] that cannot in general be exactly sampled, complicating its use numerically. However, as we saw for the AFH model numerical results, the hidden unit complexity is basis dependent. Surprisingly, we will show next that an exact M∼O(N) spin-1 NQS representation of the AKLT state is obtained in the xyz basis.

### 5.1. Exact Spin-1 NQS for AKLT State in the xyz Basis

The AKLT state provides an instructive example of how a single spin basis change can significantly alter the amplitude structure. Transforming the MPS representation into the xyz basis yields matrices

$$\begin{array}{ccc} \;B^{x} & = & \frac{1}{\sqrt{2}}\left(A^{+}-A^{-1}\right)=\frac{1}{2}{\hat{\sigma }}^{x},\;B^{y}=\frac{-i}{\sqrt{2}}\left(A^{+1}+A^{-1}\right)=\frac{1}{2}{\hat{\sigma }}^{y},\;B^{z}=-A^{0}=\frac{1}{2}{\hat{\sigma }}^{z}, \end{array}$$

and, thus, renders the amplitudes into products of Pauli matrices

$$\Psi_{\mathrm{AKLT}}\left(\alpha \right)=\mathrm{tr}\left(B^{\alpha_{1}}B^{\alpha_{2}}\cdots B^{\alpha_{N}}\right),$$

where $\alpha =(\alpha_{1},\cdots ,\alpha_{N})$ with $\alpha_{j}=\left\{x,y,z\right\}$ label the xyz basis. As expected, there is no change in the complexity/internal dimension of the MPS representation.

The structure of the amplitudes $\Psi_{\mathrm{AKLT}}\left(\alpha \right)$ in the xyz basis is significantly simpler than $\Psi_{\mathrm{AKLT}}\left(S\right)$ in the $S^{z}$ basis. Amplitudes are now evaluated by tracking the anticommutations of Pauli matrices required to make the matrices of each the type form a contiguous sequence, e.g., xx⋯xyy⋯yzz⋯z, and then reducing the product repeatedly via ${\left({\hat{\sigma }}^{\alpha }\right)}^{2}=𝟙$. The resulting matrix trace is non-zero only when the overall product is ∝𝟙, and so all non-zero amplitudes have an equal magnitude. Depending on whether *N* is even or odd, this condition requires that there is either an even or odd number of x,y and *z*’s in any configuration string, respectively. Using this, we arrive at the following result:

**Theorem** **1** (AKLT state xyz NQS). *The AKLT state in the xyz spin basis has an exact spin-1 NQS representation requiring M=2N hidden units.*

**Proof.**  We establish this result using a direct and intuitive construction for $\Psi_{\mathrm{AKLT}}\left(\alpha \right)$ in which hidden units are devised to implement the nodal structure and sign structure of this state. The rules governing the amplitudes are as follows:

1. To implement the parity constraint on the number of x,y and *z*’s in any configuration string α, we introduce the following 2×3 coupling matrices:
  $$C_{xy}=\left[\begin{array}{ccc} 1 & 1 & 1\\ -1 & -1 & 1 \end{array}\right],\;C_{yz}=\left[\begin{array}{ccc} 1 & 1 & 1\\ 1 & -1 & -1 \end{array}\right].$$
  By defining two hidden units from these matrices as $\Upsilon_{xy}=\left\{C_{xy},C_{xy},\cdots ,C_{xy}\right\}$ and $\Upsilon_{yz}=\left\{C_{yz},C_{yz},\cdots ,C_{yz}\right\}$, we arrive at the product filter
  $$\Upsilon_{xy}\left(\alpha \right)\Upsilon_{yz}\left(\alpha \right)=\left(1+{\left(-1\right)}^{\#\;x{}^{\prime }s\;+\;\#\;y{}^{\prime }s}\right)\left(1+{\left(-1\right)}^{\#\;y{}^{\prime }s\;+\;\#\;z{}^{\prime }s}\right),$$
  in which the hidden units cancel out any strings α that have odd numbers of both *x*’s and *y*’s, and *y*’s and *z*’s, respectively. Together, these hidden units completely establish for any *N* the nodal structure of the AKLT state amplitudes in this basis.
2. To reproduce the sign structure arising from anticommuting Pauli matrices into a contiguous sequence, we require two types of hidden units. The first type of hidden unit uses a conditional coupling matrix for the local state $|\;x\rangle$
  $$C_{x}=\left[\begin{array}{ccc} 0 & 1 & 1\\ 1 & 0 & 0 \end{array}\right],$$
  along with $C_{yz}$ to define a hidden unit of the form
  $$\Upsilon_{x(k)}=\left\{C_{yz},\cdots ,C_{yz},\overset{k}{\overbrace{C_{x}}},I,\cdots ,I\right\},$$
  where $C_{x}$ appears in the *k*th position in the sequence. The action of $\Upsilon_{x(k)}$ is to induce on a configuration a factor ${\left(-1\right)}^{\#\;y{}^{\prime }s\;+\;\#\;z{}^{\prime }s}$ between site k−1 and the left boundary, conditional on site *k* being in state $|\;x\rangle$. This is the sign that would occur if a $\sigma^{x}$ matrix was anticommuted to this boundary through the corresponding product of Pauli matrices. The second type of hidden unit uses two further coupling matrices
  $$C_{y}=\left[\begin{array}{ccc} 1 & 1 & 1\\ 1 & -1 & 1 \end{array}\right],\;C_{z}=\left[\begin{array}{ccc} 1 & 1 & 0\\ 0 & 0 & 1 \end{array}\right],$$
  defining a hidden unit of the form
  $$\Upsilon_{z(k)}=\left\{I,\cdots ,I,\overset{k}{\overbrace{C_{z}}},C_{y},\cdots ,C_{y}\right\},$$
  where $C_{z}$ appears in the *k*th position in the sequence. The action of $\Upsilon_{z(k)}$ is to induce on a configuration a factor ${\left(-1\right)}^{\#\;y{}^{\prime }s}$ between site k+1 and the right boundary, conditional on site *k* being in state $|\;z\rangle$. This is the sign that would occur if a ${\hat{\sigma }}^{z}$ matrix was anticommuted to the right boundary through the corresponding product of Pauli matrices, assuming that any ${\hat{\sigma }}^{x}$’s have already been anticommuted to the left boundary. To capture all locations *k* for both types, thus, requires 2(N−1) hidden units which entirely establish the sign structure of the AKLT state amplitudes in this basis.

This gives a total of M=2N hidden units. □

**Figure 7:**
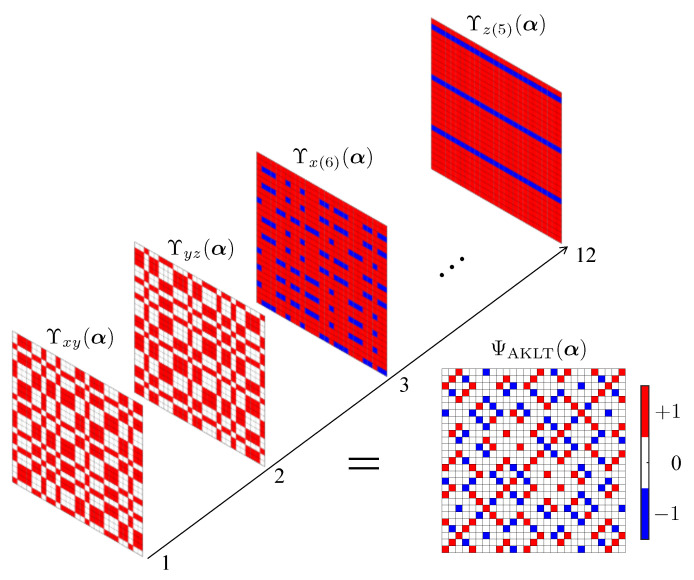
The $3^{6}$ (unnormalized) amplitudes of the N=6 AKLT state in the xyz basis $\Psi_{\mathrm{AKLT}}\left(\alpha \right)$ are displayed here as a $3^{3}\times 3^{3}$ matrix with the color of an element designating which of the values +1,0,−1 the amplitude has. The bottom left corner element corresponds to the amplitude of $|\;xxxxxx\rangle$, while the top right corner corresponds to $|\;zzzzzz\rangle$. The amplitudes $\Psi_{\mathrm{AKLT}}\left(\alpha \right)$ are reconstructed exactly by the product of M=2N=12 hidden unit filters given in the main text shown. The first two filters $\Upsilon_{xy}\left(\alpha \right)$ and $\Upsilon_{yz}\left(\alpha \right)$ establish the nodal structure, while the other ten filters $\Upsilon_{x(k)}\left(\alpha \right)$ and $\Upsilon_{z(k)}\left(\alpha \right)$ imprint the sign structure.

The resulting amplitude-wise product decomposition of the AKLT into hidden unit correlators is depicted Figure 7 for N=6.

### 5.2. Analytic Example—AKLT Unary Stabilizer State

An explicit NQS construction for the AKLT state in the xyz basis has been given before in Ref. [40] using unary encoded cells of {a,b,c} spin-$\frac{1}{2}$’s. Their construction involves initializing the b spin-$\frac{1}{2}$’s in state $|\;+\rangle$ while entangling the a and c spin-$\frac{1}{2}$’s between adjacent unary cells in the state $|\;↑\rangle |\;↑\rangle +|\;↓\rangle |\;↓\rangle$. As a tensor network, this is represented as
(30)
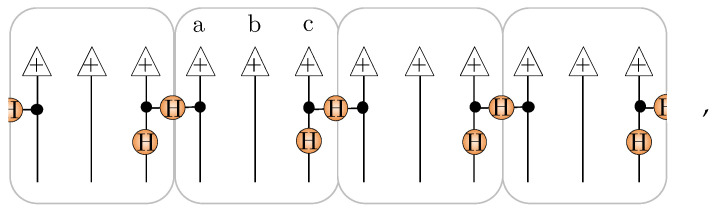

where each box is a unary cell, and H denotes the Hadamard matrix. Each unary cell then has the following unitary applied to it
(31)
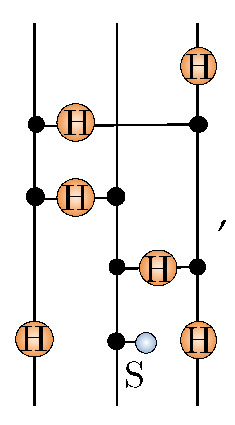

where S is the phase gate [40] (we have switched the controlled-NOT gates in the circuit given in Figure 5b of ref. [40] into controlled-*Z* gates here to expose the graph state equivalence). Putting these pieces together, the unary encoded spin-$\frac{1}{2}$ state $|\;\Psi_{\mathrm{unary}}\rangle$ is a stabilizer state constructed by the circuit
(32)
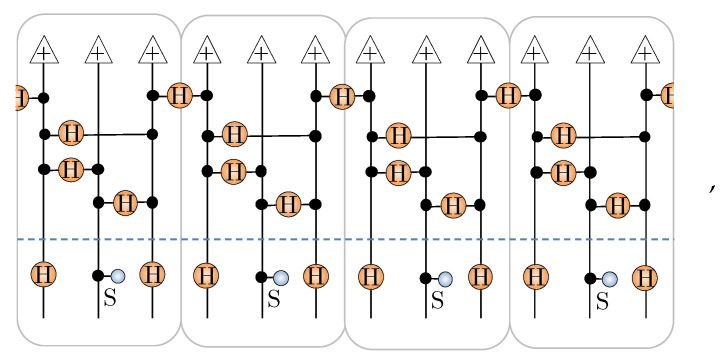

where the top part (above the dashed line) generates a graph state, and the bottom part applies local Clifford gates. Once unary projection to the spin-1 is applied, $|\;\Psi_{\mathrm{unary}}\rangle$ generates the AKLT state [40].

Previously, in ref. [42], we showed how any stabilizer state for *N* spin-$\frac{1}{2}$’s can be readily converted into an NQS with M≤N−1. Here, we just summarize the basic process. The first step in this conversion is to use the local Clifford equivalence of stabilizer states to graph states to relocate all the non-diagonal Clifford gates to independent vertices of the graph. This conversion takes the simple chain-like graph state and pattern of Clifford gates from Equation (32) and gives the following for N=12 spins:
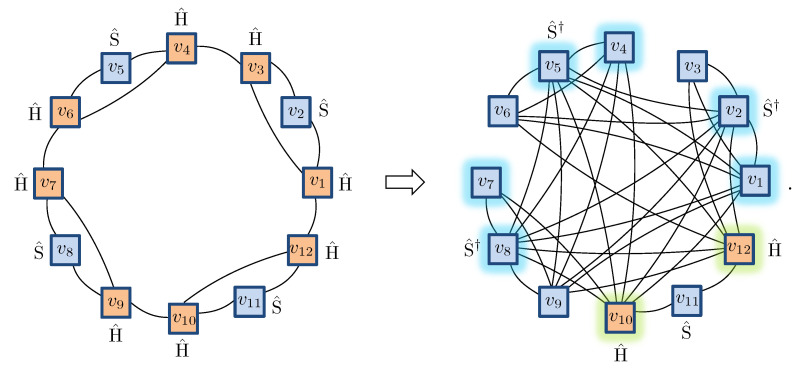
 As required, the resulting graph has diagonal Clifford gates on all vertices, except for a small independent set I={10,12} highlighted. Notice that the three-site translational invariance of $|\;\Psi_{\mathrm{unary}}\rangle$, mirrored by the initial chain graph, is still formally present in the transformed graph but is now obscured by its highly connected topology. By forming a vertex cover C={I,1,2,4,5,7,8}, we obtain a NQS [42] with M=8 hidden units (despite the more complex graph, this is the same number of hidden units required to describe the initial chain graph state as an NQS):(33)
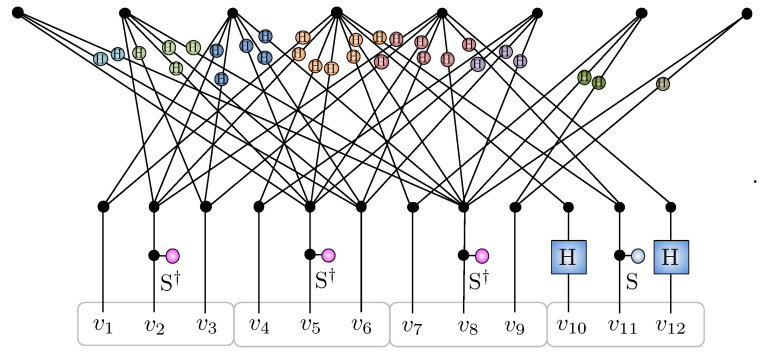
 More generally, for *N* spin-$\frac{1}{2}$’s, this procedure generates an NQS with M=2N/3 hidden units.

After applying the unary projection and contraction process directly to this spin-$\frac{1}{2}$ NQS, we obtain spin-1 NQS tensor network, whose schematic structure is shown here for N=4:(34)
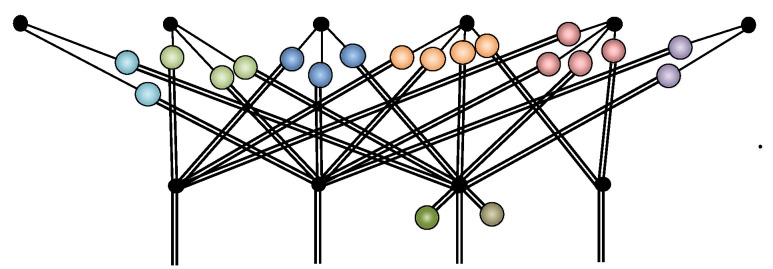
 It is evident that pairs of hidden units possess coordination 4,3 and 2, while one pair gets projected down to a spin-1 visible bias, giving M=6 overall. The same structure applies for general *N* with pairs of equal coordination spanning N,N−1,⋯,2, giving a spin-1 NQS for the AKLT state in the xyz basis requiring M=2N−2 hidden units. This representation is essentially identical to the one presented in Section 5.1, except that the hidden units implementing the nodal structure (the fully connected pair) also contribute to the sign structure, reducing the total hidden unit count by one pair. This raises an interesting question of whether an even more compressed spin-1 NQS representation of the AKLT state is possible. We finish by examining this using direct numerical optimizations.

### 5.3. Numerical Example—AKLT in xyz and $S^{z}$ Bases

Although the AKLT state is translationally invariant, the hidden units encoding the sign structure of this solution are neither individually translationally invariant nor do their translates appear. Consequently, we performed VMC calculations with increasing *M* using both the spin-1 NQS and unary NQS for N={6,8,10,12} and compared against exact diagonalization. As with the AFH model (which is ${\hat{H}}_{\mathrm{AKLT}}$ with β=0), earlier in Section 4.5, we considered both the $S^{z}$ and xyz basis with their corresponding nodal structure enforced by sampling. We also utilize the same sequential growth scheme as we used in the Heisenberg calculations, again confirming the robustness of our qualitative conclusions by performing reruns of the optimization sequence and presenting the best results here.

**Figure 8:**
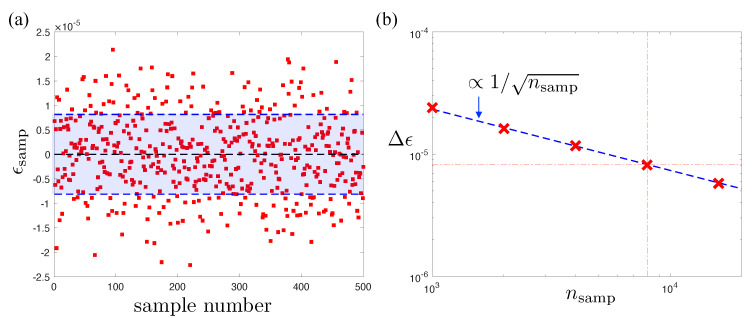
(**a**) The energy $\epsilon_{\mathrm{samp}}$ (red squares) for 500 independent Monte Carlo runs of the N=12 exact AKLT spin-1 NQS each consisting of $n_{\mathrm{samp}}=8000$ individual sampling steps separated by *N* individual Markov chain moves to reduce autocorrelation effects. The standard deviation Δϵ for these samples around the exact zero ground state energy is shown by the blue band. (**b**) The standard deviations Δϵ of energies sampled versus the number of samples $n_{\mathrm{samp}}$ used. Each point is also calculated from 500 independent samples, and the number of sampling steps per evaluation $n_{\mathrm{samp}}=\{1000,2000,4000,8000,16$,000}. The fluctuations in energy closely follow a $1/\sqrt{n_{\mathrm{samp}}}$ scaling (dashed line), as expected for a Monte Carlo sampling process [30]. The red dotted lines are to guide the eye to the point for $n_{\mathrm{samp}}=8000$, which is representative of an optimization step for our VMC calculations.

As we are performing stochastic optimization, intrinsic sampling noise will limit the accuracy to which any formally exact solution can be found. It is, therefore, crucial to quantitatively characterize when exactness may have been reached numerically. For a gapped Hamiltonian, the average energy of an approximate state *E* can be related to its infidelity with the ground state using [30]

(35) $$1-O\leq \frac{E-E_{0}}{\delta }=\frac{\epsilon }{\delta },$$

where δ is the energy gap of the Hamiltonian, and $\epsilon =E-E_{0}$ is the energy deviation. As the ground state energy $E_{0}$ of the AKLT Hamiltonian is zero, the energy deviation is simply the sampled energy of the state $\epsilon_{\mathrm{samp}}$. As shown in Figure 8a, even if the exact analytic spin-1 NQS solution is used, $\epsilon_{\mathrm{samp}}$ fluctuates when using a finite number of samples typical of an optimization step. For all optimizations presented in this paper, we used the following hyperparameters: number of samples per optimization step $n_{\mathrm{samp}}=8000$, number of optimization steps $n_{\mathrm{step}}∼5000$. Typically, the full wavefunction and its fidelity are calculated and checked every 1000 steps to gauge whether the solution has converged or requires further optimization. To account for fluctuations caused by a finite $n_{\mathrm{samp}}$ employed throughout the stochastic optimization, therefore, we use, in Equation (35), ϵ=Δϵ, the standard deviation of the sampled energy. As shown in Figure 8b, Δϵ vanishes as $1/\sqrt{n_{\mathrm{samp}}}$, and we estimate an algorithmic fidelity resolution of $R=\Delta \epsilon /\delta \approx 1.2\times 10^{-5}$ for N=12 sites below, in which it may be hard to discriminate an exact solution from an extremely good approximate one.

**Figure 9:**
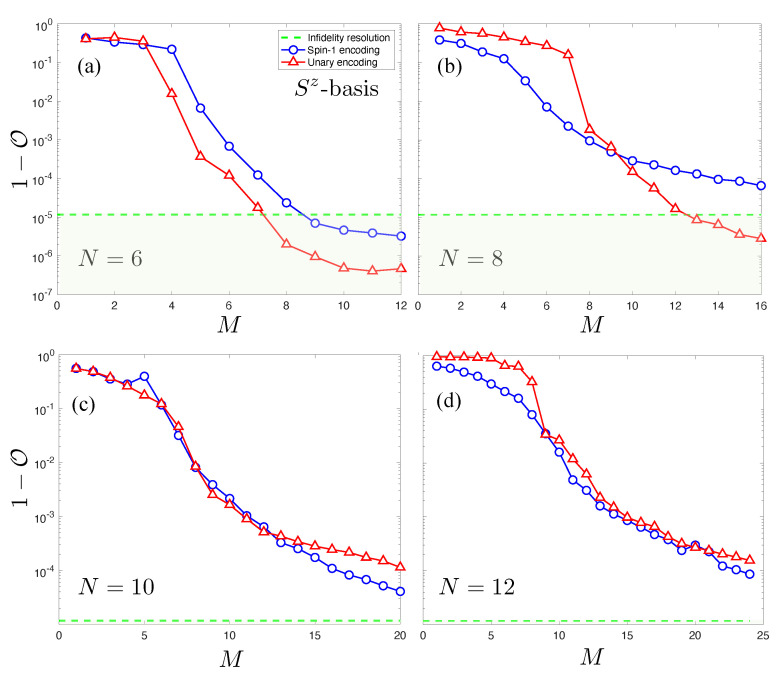
Plots of the infidelity of unary (red stars) and spin-1 (blue squares) NQS with the AKLT state in the $S^{z}$ basis. The infidelity bound R is also plotted as a dotted green line. Results for four system sizes are plotted: (**a**) N=6, (**b**) N=8, (**c**) N=10, (**d**) N=12.

In Figure 9, we show the smooth decrease in 1−O against *M* for the $S^{z}$ basis. While the N=6 curve in Figure 9a drops below R, this is not attained for larger *N* shown in Figure 9b–d within the *M*’s tested. This indicates that no “exact” NQS solutions have been located. As with the AFH model, the spin-1 NQS and unary NQS achieve a similar accuracy verses *M*, although the former utilizes less variational parameters.

**Figure 10:**
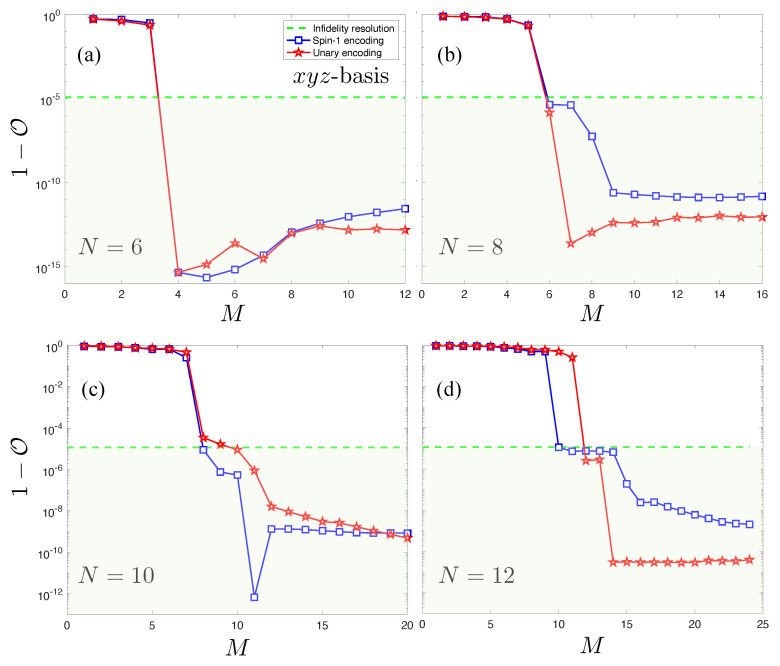
Plots of the infidelity of unary (red stars) and spin-1 (blue squares) NQS with the AKLT state for the xyz basis. The infidelity resolution R is plotted as a green dotted line. Results for four system sizes are plotted: (**a**) N=6, (**b**) N=8, (**c**) N=10, (**d**) N=12.

The analogous results in Figure 10 for the xyz basis display remarkable features in comparison. For each *N*, a sharp drop in 1−O by over 4 orders of magnitude is observed at M=N−2 that consistently pushes the infidelity below R. After reaching this point, 1−O versus *M* plateaus, and subsequent hidden units have negligible bearing on the accuracy of the wavefunction due to statistical fluctuations originating from the stochastic optimization. These features are consistently produced by both the spin-1 NQS and unary NQS, aside from the largest system size N=12 in Figure 10d, indicating that the increasing number of redundant variational parameters in the unary encoding is complicating the optimization. The overall behavior of 1−O observed in this basis is strong evidence of a numerically exact solution with M=N (once the 2 hidden units implementing the nodal structure are included). This is substantially smaller than the analytic solutions introduced and is very suggestive of there being a *compact* exact spin-1 NQS representation of the AKLT state in the xyz basis.

## 6. Conclusions

We have introduced the most natural and direct generalization of RBM from spin-$\frac{1}{2}$ to spin-1. This necessitated including a quadratic visible bias and a quadratic visible-hidden interaction in the RBM energy function to ensure trivial product state representation, labeling freedom and gauge equivalence to the tensor network formulation. We demonstrated its use numerically for the spin-1 AFH model in both the $S^{z}$ and xyz bases, illustrating how the choice of basis can affect the accuracy and hidden unit complexity of an NQS representation. Using our spin-1 NQS, we then re-examined how to represent the AKLT state exactly. In the $S^{z}$ basis, it is known to require $M∼O(N^{2})$ hidden units, yet, by changing to the xyz basis, we construct an NQS with M∼O(N) hidden units.

Numerical VMC calculations have indicated that, by capturing the nodal structure, either implicitly within the sampling or explicitly through the inclusion of extra hidden units, the optimization can find even more efficient constructions for the sign structure. The resulting spin-1 NQS for the AKLT state in the xyz basis requires M=N hidden units in total making it *compact*. This example raises the interesting possibility of improving the efficiency and accuracy of NQS calculations by including single-spin basis transformations to lower the hidden unit complexity.

Several important open questions remain about NQS representations. In particular, it would be instructive to build representations of classes of bosonic states using multinomial RBMs. In this case, a local Fock basis is typically employed; however, our findings suggest that it could be useful to explore a local basis that breaks the particle number symmetry when describing condensates. Moreover, the elevation of visible units from binary to multinomial raises the question of whether also using multinomial hidden units can enhance the expressiveness of NQS. This has been explored in the context of binary visible units in ref. [54] in an analytical context to precisely represent certain two- and three-body interactions. The use of multinomial hidden units for numerical VMC calculations has been largely unexplored and is the subject of forthcoming work [55] for the Bose Hubbard model in two dimensions.   

## Data Availability

MATLAB scripts and .mat files containing the data shown in the figures are available in the data repository ref. [56], doi:10.5523/bris.1ln9kyt6i86n12ehhftht27edp.

## Appendix A. Monte Carlo Method and Sampling

Any many-body quantum state can be expressed in terms of its amplitudes on a set of configurations states in a chosen basis, but exhaustively computing the amplitudes for every possible configuration in the basis is often prohibitively expensive. Monte Carlo methods circumvent this by randomly selecting a subset of these configurations, and calculating an estimate of key observables using this selection. In this case, we require only that the (unnormalized) amplitudes $\Psi_{p}\left(S\right)$ of the ansatz (defined by parameters p) in a fixed basis, such as $S^{z}$, can be computed in a time with polynomial complexity in *N*. We then rewrite the expectation value as

(A1) $${\langle \hat{A}\rangle }_{\Psi_{p}}=\sum_{S}P\left(S\right)A\left(S\right),\;\mathrm{where}\;P\left(S\right)=\frac{|\Psi_{p}{\left(S\right)|}^{2}}{\sum_{S}{\left|\Psi_{p}\left(S\right)\right|}^{2}}$$

is the probability of the spin configuration S, and

(A2) $$A\left(S\right)=\sum_{S^{\prime }}\frac{\Psi_{p}\left(S^{\prime }\right)}{\Psi_{p}\left(S\right)}\langle S\;|\hat{A}|\;S^{\prime }\rangle$$

is the local estimator of $\hat{A}$. The sum over $S^{\prime }$ in Equation (A2) is restricted to only those configurations for which the matrix element $\langle S\;|\hat{A}|\;S^{\prime }\rangle \neq 0$, and, so as long as $\hat{A}$ is sparse in the chosen fixed basis, then A(S) can be efficiently evaluated. Typical product terms comprising short-ranged Hamiltonians $\hat{H}$ fulfill this requirement. Thus, an unbiased estimate of ${\langle \hat{A}\rangle }_{\Psi_{p}}$ is made by drawing independent samples of spin configurations S from the distribution P(S). A powerful way to accomplish this is Markov-chain Monte Carlo in which a sequence of spin configurations $S_{0}\to S_{1}\to \cdots$ is generated using the Metropolis-Hastings algorithm that accepts a proposed configuration $S_{\mathrm{prop}}$ for the *n*th iteration with a probability $P_{n}\left(S_{\mathrm{prop}}\right)=\min[1,|\Psi_{p}\left(S_{\mathrm{prop}}\right){|}^{2}/|\Psi_{p}\left(S_{n-1}\right){|}^{2}]$. A subset of visited configurations in the sequence, suitably separated to ensure they are decorrelated, is then used to estimate of ${\langle \hat{A}\rangle }_{\Psi_{p}}$. Crucially, both A(S) and Monte Carlo sampling rely only on the ratio of ansatz amplitudes, so the intractable state normalization $\sum_{S}{\left|\Psi_{p}\left(S\right)\right|}^{2}$ is entirely avoided.

Variational Monte Carlo proceeds by evaluating the ansatz energy $E_{p}={\langle \hat{H}\rangle }_{\Psi_{p}}$ and its variance, along with their gradient vectors with respect to parameters p. The parameters can then be updated by a small step along the direction of steepest descent, with this iterated until convergence to the minimum $p_{0}$ [29]. The efficiency of this minimization process is strongly problem and ansatz dependent. In challenging cases, more sophisticated approaches, such as modified stochastic optimization [57], the ‘linear method’ [58,59], and stochastic reconfiguration [30,49], are needed. All numerical calculations in this paper employ stochastic reconfiguration. The method involves the construction of a $n_{\mathrm{params}}\times n_{\mathrm{params}}$ matrix, requiring $O(n_{\mathrm{samp}}n_{\mathrm{params}}^{2})$ operations, though with a conjugate gradient method this can be brought down to $O(n_{\mathrm{samp}}n_{\mathrm{params}})$ [60]. The parameter changes can then be calculated from a set of linear equations involving the matrix, which scales as $O(n_{\mathrm{params}}^{2})$ using matrix-vector product methods. As a rule of thumb, the matrix requires a number of samples $n_{\mathrm{samp}}>10n_{\mathrm{params}}$ to ensure the sampled matrix is not rank-deficient [30], giving an overall minimum scaling of $O(n_{\mathrm{params}}^{2})$ for the algorithm, though, typically, it is prudent to have a large number of samples $n_{\mathrm{samp}}\gg n_{\mathrm{params}}$ as the error of any sampled observable, typically scales, as $1/\sqrt{n_{\mathrm{samp}}}$ [30], including the elements of the matrix.

## Appendix B. Boltzmann Parameterization of Coupling Matrices

Every 2×3 coupling matrix within the spin-1 NQS tensor network can be parameterized in Boltzmann form using the generalized energy function introduced in Section 4.2 as

(A3) $$C_{h_{i}v_{j}}^{\left(ij\right)}=\exp\left({\tilde{c}}_{ij}+w_{ij}h_{i}v_{j}+W_{ij}h_{i}v_{j}^{2}+{\tilde{b}}_{ij}h_{i}+\tilde{a_{ij}}v_{j}+{\tilde{A}}_{ij}v_{j}^{2}\right),$$

for $h_{i}\in \left\{+1,-1\right\}$ and $v_{j}\in \left\{+1,0,-1\right\}$, which includes a quadratic weight $W_{ij}$ and quadratic partial bias ${\tilde{A}}_{ij}$. These complex parameters are found from the coupling matrix elements as

$$\begin{array}{c} {\tilde{a}}_{ij}=\frac{1}{4}\left[\log\left(C_{++}^{\left(ij\right)}\right)+\log\left(C_{-+}^{\left(ij\right)}\right)-\log\left(C_{+-}^{\left(ij\right)}\right)-\log\left(C_{--}^{\left(ij\right)}\right)\right],\\ {\tilde{b}}_{ij}=\frac{1}{2}\left[\log\left(C_{+0}^{\left(ij\right)}\right)-\log\left(C_{-0}^{\left(ij\right)}\right)\right],\\ {\tilde{c}}_{ij}=\frac{1}{2}\left[\log\left(C_{+0}^{\left(ij\right)}\right)+\log\left(C_{-0}^{\left(ij\right)}\right)\right],\\ w_{ij}=\frac{1}{4}\left[\log\left(C_{++}^{\left(ij\right)}\right)-\log\left(C_{-+}^{\left(ij\right)}\right)-\log\left(C_{+-}^{\left(ij\right)}\right)+\log\left(C_{--}^{\left(ij\right)}\right)\right],\\ W_{ij}=\frac{1}{4}\left[\log\left(C_{++}^{\left(ij\right)}\right)-\log\left(C_{-+}^{\left(ij\right)}\right)-2\log\left(C_{+0}^{\left(ij\right)}\right)+2\log\left(C_{-0}^{\left(ij\right)}\right)+\log\left(C_{+-}^{\left(ij\right)}\right)-\log\left(C_{--}^{\left(ij\right)}\right)\right],\\ {\tilde{A}}_{ij}=\frac{1}{4}\left[\log\left(C_{++}^{\left(ij\right)}\right)+\log\left(C_{-+}^{\left(ij\right)}\right)-2\log\left(C_{+0}^{\left(ij\right)}\right)-2\log\left(C_{-0}^{\left(ij\right)}\right)+\log\left(C_{+-}^{\left(ij\right)}\right)+\log\left(C_{--}^{\left(ij\right)}\right)\right]. \end{array}$$

In both cases, the presence of zero matrix elements in $C^{\left(ij\right)}$, which appear frequently in our ‘by hand’ constructions, return diverging parameters. However, as discussed in ref. [42], this can be handled by softening the zeros via

(A4) $$C_{h_{i}v_{j}}^{\left(ij\right)}\mapsto \max\left(C_{h_{i}v_{j}}^{\left(ij\right)},e^{-S}\right),$$

where typically S≈ 5–10.

## Appendix C. AKLT State NQS in S z Basis

In this appendix, we construct a spin-1 NQS for the AKLT state amplitudes in the $S^{z}$ basis given in Equation (26) by using hidden units as successive filters. The rules governing the structure of the amplitudes can be summarized as follows:

1. Zero out the amplitude for any configuration containing a substring for any ℓ=0,1,⋯,N−2 of the form $+{\left[0\cdots 0\right]}^{\ell }+$ or $-{\left[0\cdots 0\right]}^{\ell }-$, where ${\left[0\cdots 0\right]}^{\ell }$ is a string of 0’s of length *ℓ*. To implement this rule, we introduce the following 2×3 coupling matrices:
  $$\;C_{i⇑}=\left[\begin{array}{ccc} 1 & 1 & 1\\ i & 0 & 0 \end{array}\right],\;C_{i⇓}=\left[\begin{array}{ccc} 1 & 1 & 1\\ 0 & 0 & i \end{array}\right],\;C_{+0}=\left[\begin{array}{ccc} 1 & 1 & 1\\ 0 & 1 & 0 \end{array}\right],$$
  which, in turn, single out $S^{z}$ basis spin-1 states $|\;⇑\rangle$, $|\;0\rangle$ and $|\;⇓\rangle$. To cancel out in any configuration substrings $+{\left[0\cdots 0\right]}^{\ell }+$ and $-{\left[0\cdots 0\right]}^{\ell }-$, starting at a given site *k* with a given separation *ℓ*, we construct pairs of hidden units with
  $$\begin{array}{c} \{I,\cdots ,I,\overset{k}{\overbrace{C_{+i}}},C_{+0},\cdots ,C_{+0},\overset{k+1+\ell }{\overbrace{C_{+i}}},I,\cdots ,I\},\;\mathrm{and}\\ \{I,\cdots ,I,\overset{k}{\overbrace{C_{-i}}},C_{+0},\cdots ,C_{+0},\overset{k+1+\ell }{\overbrace{C_{-i}}},I,\cdots ,I\}. \end{array}$$
  To capture all separations ℓ=0,1,⋯,N−2 starting on any site k=1,2,⋯,N, we require 2N(N−1) hidden units.
2. Apply a factor of ${\left(-1\right)}^{\ell }$ to the amplitude of a configuration for each substring of the form $+{\left[0\cdots 0\right]}^{\ell }-$ it contains. To implement this rule, we introduce two additional coupling matrices:
  $$C_{2⇑}=\left[\begin{array}{ccc} 1 & 1 & 1\\ 2 & 0 & 0 \end{array}\right],\;C_{-⇓}=\left[\begin{array}{ccc} 1 & 1 & 1\\ 0 & 0 & -1 \end{array}\right].$$
  A factor ${\left(-1\right)}^{\ell }$ is induced on any configuration containing the substring $+{\left[0\cdots 0\right]}^{\ell }-$, starting at a given site *k* with a given separation *ℓ*, by a hidden unit with
  $$\{I,\cdots ,I,\overset{k}{\overbrace{C_{2⇑}}},C_{+0},\cdots ,C_{+0},\overset{k+1+\ell }{\overbrace{C_{-⇓}}},I,\cdots ,I\}.$$
  To capture all odd separations ℓ=1,3,⋯,N−2 starting on any site k=1,2,⋯,N requires $N\lfloor \frac{1}{2}\left(N-1\right)\rfloor$ hidden units.
3. Zero out the single excitation configurations $+{\left[0\cdots 0\right]}^{N-1}$ and $-{\left[0\cdots 0\right]}^{N-1}$ and all their translates. To implement this rule, we introduce another coupling matrix:
  $$C_{-⇑}=\left[\begin{array}{ccc} 1 & 1 & 1\\ -1 & 0 & 0 \end{array}\right].$$
  Configurations $+{\left[0\cdots 0\right]}^{N-1}$ and $-{\left[0\cdots 0\right]}^{N-1}$ are then cancelled out by a pair of hidden units with $\left\{C_{-⇑},C_{+0},\cdots ,C_{+0}\right\}$ and $\left\{C_{-⇓},C_{+0},\cdots ,C_{+0}\right\}$. Capturing all the translates is achieved with 2N hidden units.
4. For *N*, odd zero out the amplitude for configuration ${\left[0\cdots 0\right]}^{N}$, or, for *N*, even double its amplitude. To implement this rule, we introduce one final coupling matrix:
  $$C_{-0}=\left[\begin{array}{ccc} 1 & 1 & 1\\ 0 & -1 & 0 \end{array}\right],$$
  and defining a single hidden unit as $\left\{C_{-0},C_{-0},\cdots ,C_{-0}\right\}$.
5. Apply a factors of $2^{\left(\#\;+{}^{\prime }s\right)}\times {\left(-1\right)}^{\left(\#\;0{}^{\prime }s\right)}\times {\left(-1\right)}^{\left(\#\;-{}^{\prime }s\right)}$ to the amplitudes of all configurations. This does not require any additional hidden units. Instead, we can pick any hidden unit and right multiply each of its *N* coupling matrices by D=diag(2,−1,−1). In RBM formalism, this is equivalent to setting non-zero visible unit biases.

This gives a total of $M=2N^{2}+N\left\lfloor \frac{1}{2}\left(N-1\right)\right\rfloor +1$ hidden units. Notice that the origin of the $O(N^{2})$ scaling arises from spatial translations, so if this symmetry is directly imposed within the NQS ansatz, as it often is numerically, then the AKLT state in the $S^{z}$ basis instead has its number of unique hidden units scaling as $M=2N+\lfloor \frac{1}{2}\left(N-1\right)\rfloor +1$.
